# Face-to-trait inferences in Japanese children and adults based on Caucasian faces

**DOI:** 10.3389/fpsyg.2022.955194

**Published:** 2022-12-07

**Authors:** Yuiko Sakuta

**Affiliations:** Faculty of Human Life Sciences, Jissen Women’s University, Tokyo, Japan

**Keywords:** face-to-trait inference, development, impression, cultural differences, trustworthiness

## Abstract

Recently, many studies have indicated that humans make social evaluations from facial appearances instantaneously and automatically. Furthermore, such judgments play an important role in several social contexts. However, the mechanisms involved in the ability to form impressions from faces are still unknown, as is the extent to which these can be regarded as universal in perceiving impressions. In the current study, computer-generated Caucasian faces were used to assess the universality or cultural differences in impression formation among Japanese children and adults. This study hypothesized that impressions of trustworthiness and dominance may be more fundamental and universal, whereas the impression of competence may be more complex and culture-dependent. In Experiment 1a, 42 children aged 3–6 years were presented with 10 pairs of face images and asked which image in each pair was more trustworthy, dominant, or competent. Overall, it was found that as age increased, the rate of agreement of Japanese participants with the judgment of American participants, obtained in a previous study, increased. However, the agreement rate for competence was relatively low. Experiment 1b, conducted with 46 children, was a replication of Experiment 1a, and the results showed the same tendency. In Experiment 2, 45 Japanese adults made impression judgments on 19 pairs of face images identical to those used in Experiment 1b. The results suggested that while dominance was a dimension not easily influenced by developmental changes or culture, trustworthiness could be influenced by cultural differences in facial expression recognition. Therefore, different judgment criteria are used for children and adults. For competence, the agreement rate with Americans was relatively stable and low among the different age groups. This suggests that depending on the dimension of the trait, certain judgments are influenced by cultural context and, therefore, change criteria, while others are based on more universal criteria.

## Introduction

When we see a person, we often rapidly draw inferences regarding their traits, such as trustworthiness or competence. Recently, various studies have clarified that humans can immediately make social evaluations from facial appearance and that such judgments play an important role in several social contexts. The ability to rapidly detect a trustworthy person is especially important for forming good relationships within a social community.

Trait inference from a face occurs automatically, spontaneously, and rapidly (Willis and Todorov, 2006; Todorov et al., 2009). Todorov et al. (2009) demonstrated that it even occurred within 50 ms after exposure to a face and that trustworthiness influenced priming, even when the face was presented subliminally. Furthermore, various studies clarified that face-to-trait inference or impression judgment played an important role in several social contexts. For example, it can affect the outcome of the U.S. congressional elections (Todorov et al., 2005; Rule et al., 2010). Judgments regarding a candidate’s competency correlate strongly with judgments regarding their facial attractiveness, which in turn can correctly predict approximately 70% of congressional electoral outcomes (Todorov et al., 2005). Thus, first impressions based on facial appearance unconsciously affect our choices or judgments in various social settings.

In social psychology, “warmth” and “competence” are two important decision axes in interpersonal impressions (Fiske et al., 2007; Cuddy et al., 2008). People in various occupations and positions in society are distributed in a two-dimensional space based on a combination of warmth and competence parameters. Oosterhof and Todorov (2008) also proposed trustworthiness and dominance as basic dimensions through a principal component analysis (PCA) as described below.

Recently, data-driven approaches have attracted attention. Oosterhof and Todorov (2008) had participants infer characteristics from faces and applied a PCA to reduce characteristic judgments to two basic dimensions: trustworthiness/valence and dominance. They collected trait ratings for 300 computer-generated faces to cross-validate their model. The principal components of the physical attributes of these faces were provided. Oosterhof and Todorov (2008) mapped the perceived characteristic dimensions onto the “face” defined by these physical dimensions, centered on an average face [based on a procedure by Blanz and Vetter (1999)]. In this model, faces higher than the average of the trustworthiness dimension appeared to smile, while those lower appeared to be increasingly angry. Dominant faces appeared more mature, masculine, and darker (Oosterhof and Todorov, 2008; Todorov and Oosterhof, 2011). Furthermore, the faces generated to be placed on these two dimensions were actually perceived to differ in trustworthiness and dominance by new participants (Oosterhof and Todorov, 2008). A similar approach was proposed by Sakuta et al. (2009). Sakuta et al. (2009) extracted factors that roughly matched the three factors by Osgood et al. (1957). Sutherland et al. (2013) used various face images, which included a wide range of ages, expressions, and poses, and found an additional dimension, “youthful-attractiveness,” which related increasing age to perceptions of decreasing attractiveness, and extended model Oosterhof and Todorov (2008).

As visualized by Oosterhof and Todorov (2008), impression judgments from faces are considered to be a generalization of emotion perception. Happy and angry facial expressions increase and decrease trustworthiness judgments, respectively (Oosterhof and Todorov, 2008; Todorov et al., 2008; Sutherland et al., 2017). Even when presented with neutral faces, adults are more likely to use subtle expressive signs of positive or negative affect to judge trustworthiness, a phenomenon known as emotional overgeneralization (Todorov, 2008; Zebrowitz, 2017). In other words, previous findings indicated that adults tended to trust individuals who expressed positive rather than negative emotions, and did so even when the expressive cues were subtle. Furthermore, Verosky and Todorov (2013) showed that participants rated new faces that resembled familiar negative faces more negatively than those that resembled familiar positive faces. The authors stated that generalization learning based on the physical similarity of faces was a powerful and relatively automatic process that could influence face ratings in various situations. In addition, Lee et al. (2021) used artificial object images (greebles) in an experiment with adults to examine whether the origin of first impressions was due to learning. Results showed that first impressions from appearance were rapidly learned, and that once learned, they were detectable in a very short time. This study suggests that first impressions are not necessarily innate. The current study focuses on the development and cultural differences on impression perception.

Several studies have reported cultural universality in the trait-inference from faces (e.g., Zebrowitz et al., 1993, 2012; Cunningham et al., 1995; Rule et al., 2010, 2011), while others reported both universalities and some differences between cultures (Walker et al., 2011; Sutherland et al., 2018). Particularly, the finding that the Tsimane people, who live isolated in the Bolivian rainforest, and American raters generally agreed on facial impressions is noteworthy (Zebrowitz et al., 2012). The cross-cultural similarity in the characteristics of impressions received from faces has been considered to be partially influenced by culture-specific perceptual learning, although there may be universal mechanisms at work. Sutherland et al. (2018) built a data-driven model with Chinese and British people, and found common “approachability” and strong evidence for the “youthfulness” dimension, as well as evidence for a third dimension similar to “competence.” Impressions along these dimensions were thought to be based on adaptive cues for threat detection and sexual selection, and this finding was strong evidence that facial impressions were shared across cultures.

In contrast, many studies have shown cultural differences in face perception. For example, several studies that measured eye gaze during face observation found cultural differences in gazing behavior. For example, in Blais et al. (2008), Western Caucasian observers showed a triangular gaze pattern, which consisted of two eyes and a mouth for both races’ faces, for all tasks. Conversely, East Asians gazed more at the center of the face. These results indicated that face processing could not be considered universal. Jack et al. (2009) also conducted a gaze measurement experiment and found that Eastern observers gazed relentlessly at the eye area instead of evenly across the face as Westerners did. The authors also pointed out that the strategies and categories of facial expression recognition differed by culture.

Recent studies have pointed out the possibility that infants perceive impressions similar to adults to an extent. Cogsdill et al. (2014) verified that 3-year-old children made impression judgments in a manner similar to adults. Such judgments became more stable at 5 years and older. Recent studies revealed that even infants detected trustworthiness in faces (Jessen and Grossmann, 2016, 2019; Sakuta et al., 2018). Thus, young children could have sensitivity for facial features that evoke facial impression, especially trustworthiness. Cogsdill and Banaji (2015) used face images of adults, children, and macaques to examine the agreement between characteristic inferences from child and adult faces. An agreement was found between child and adult judgments for all face stimuli. From these results, it was concluded that trait inferences from faces were acquired early in childhood. However, it is still unknown how the ability to perceive impressions from faces develops in very young ages, how the impressions are related to facial expressions, and whether the impressions are influenced by culture.

As noted above, first impressions have been reported to be influenced by subtle facial expressions in adults; however, there are conflicting findings in children. According to previous studies, such as Ewing et al. (2019) and Tang et al. (2019), young children’s trust judgment was influenced by facial expressions. In contrast, Mondloch et al. (2019) reported that, unlike adults, there was no evidence that children aged 4–11 years used facial expressions when forming impressions. Furthermore, it was noted that the relationship between facial expressions and first impressions was similar for children as for adults in explicit impression judgment tasks. However, no consistent findings were available for tasks that involved implicit processing (Van Der Zant et al., 2021).

Thus, research on impression perception in infancy has received increasing attention recently. However, much remains unknown. To approach cultural differences, a detailed examination of impression perception during infancy, when the influence of culture and experience is still small, may be an effective method.

In the current study, I used computer-generated Caucasian faces to assess the universality of perceiving facial impressions in Asian (Japanese) children. Previous studies have shown that the other-race effect in infants occurs at around 6 months of age and is present at around 9 months of age (Pascalis et al., 2005; Kelly et al., 2007). However, these previous studies were only concerned with face identification and not with impression consensus. Therefore, it cannot be said that other-race effects also occur in infants’ impression perception. Japanese infants aged 6–8 months have been found to gaze at faces deemed trustworthy by American adults in experiments with Caucasian faces (Sakuta et al., 2018). Thus, it is possible that at least infants were able to detect some trait that leads to trustworthiness, regardless of the race of the face. Moreover, one of the cues for trust detection is a facial expression that looks like a smile (e.g., raised corners of the mouth), and it is possible that American adults and Japanese infants alike use “raised corners of the mouth” as a cue to perceive trustworthiness. However, the facial stimuli used in this study and in Sakuta et al. (2018) were originally created with neutral expressions and do not show a clear smile, although the faces with high trustworthiness appear to be smiling slightly. It should be noted that at around 6–7 months, infants cannot perceive subtle facial expressions without movement (Ichikawa et al., 2014). Hence, it is possible that the facial images used in the current and Sakuta et al. (2018) studies were not perceived as smiles by the infants. Therefore, it may be necessary to consider cues other than facial expressions in infants’ impression perception. To examine whether the impression perceptions in Sakuta et al.’s (2018) were found only in infants or also in older children, it was necessary to use the same stimuli as in Sakuta et al. (2018). Testing whether infants are able to judge impressions on faces of other races and comparing the judged impressions with results obtained in other countries will be important for elucidating the mechanisms of impression perception.

According to previous studies (Oosterhof and Todorov, 2008; Cogsdill et al., 2014), it is possible that there could be both universality and some cultural differences based on the kind of impressions. In Oosterhof and Todorov’s (2008), trustworthiness was treated as a dimension independent of dominance. Results of the PCA for trait judgment showed that the first principal component, trustworthiness, had high loadings for adjectives, such as trustworthy, emotionally stable, responsible, and sociable, while the second principal component, dominance, had high loadings for dominant, aggressive, and confident. Cogsdill et al. (2014) also addressed trustworthiness as a basic evaluation and dominance and competence as more specific traits. Considering the above, trustworthiness and dominance could be more fundamental and universal, whereas competence could be more complex and culture-dependent.

Focusing on one’s appearance is an adaptive ability that helps us rapidly detect a friend or foe in a social environment. Judgment of trustworthiness can predict an individual’s social and economic success (Todorov, 2008; van ‘t Wout and Sanfey, 2008). Judgment of competence is important for choosing a leader in adults and children (Todorov et al., 2005; Antonakis and Dalgas, 2009). Todorov et al. (2008) noted that evaluating faces on valence (or trustworthiness) and dominance may be an overgeneralization of adaptive mechanisms, which attempts to estimate others’ behavioral intention and status in a hierarchy of power. The current study chose stimuli that varied on the dimensions of trustworthiness, dominance, and competence as these were important social dimensions, at least for adults. It is worth examining whether these are important for young children as well.

To examine what facial impressions were perceived in Asian people, Experiment 1a assessed Japanese children’s judgment of computer-generated Caucasian faces. Experiment 1b was conducted with another group of Japanese children to validate the results of Experiment 1a. Lastly, Japanese adults’ impression judgment was assessed in Experiment 2.

## Experiment 1a: Impression formation in Japanese children

Experiment 1a intended to replicate the prior studies regarding impression formation in children (Cogsdill et al., 2014; Sakuta et al., 2018).

### Method

#### Participants

This study included 42 children (23 girls and 19 boys) divided into two age groups: 3–4-year-olds (*n* = 25, range: 2 years 11 months–4 years 10 months) and 5–6-year-olds (*n* = 17, range: 5 years 4 months–6 years 10 months). The experiment was conducted from February to March 2017. Previous studies have shown that toddlers exhibit the face inversion effect at about 6 years of age and develop holistic processing in face recognition similarly to adults (Brace et al., 2001). Therefore, comparing the developmental process of face recognition by dividing the children into younger and older groups is possible. All the children were Japanese and recruited from a nursery school. The following experiments (Experiments 1a, 1b, and 2) were approved by the Ethical Committee of Jissen Women’s University (H28-18). Moreover, the experiments were conducted according to the principles laid down in the Declaration of Helsinki. Written informed consent was obtained from each child’s parents prior to their participation. A sample size was determined based on previous research (Oosterhof and Todorov, 2008), where approximately 20 participants made impression judgments in each study.

#### Stimuli

We used computer-generated face images, in which trustworthiness, dominance, and competence were manipulated, as stimuli. Face images were obtained from a database created in FaceGenModeller 3.2 (Singular Inversions)^1^, validated (Oosterhof and Todorov, 2008), and open to the public^2^. These were created based on data-driven, computational models (derived from adults’ judgments in the U.S.) of the respective traits (Oosterhof and Todorov, 2008). Oosterhof and Todorov (2008) proposed a two-dimensional (2D) model of face evaluation, which consisted of two orthogonal axes of valence (trustworthiness) and dominance. These two axes were composite factors obtained from a PCA: valence evaluation included positive judgments of attravvctiveness and responsibility and dominance evaluation consisted of judgments of dominance, aggressiveness, and confidence. These axes were the most important dimensions in social judgment, yet other social evaluations were also representable in this 2D model. All faces were bald, Caucasian males.

The stimuli were chosen from two databases: (A) Distinct face identities manipulated on face shape and reflectance, on several dimensions. The current study used the faces with manipulation −3SD and +3SD on competence, dominance, and trustworthiness dimensions. These databases were validated by Todorov et al. (2013). Cogsdill et al. (2014) used this type of stimuli to assess American children’s impression formation. (B) The average face was manipulated on face shape and orthogonally on perceived trustworthiness and dominance, parametric face manipulation that ranged from −3 to +3SD. The current study used the faces with manipulation −2SD and +2SD. These orthogonal models were created by Oosterhof and Todorov (2008). Sakuta et al. (2018) used this type of stimuli to assess Japanese infants’ impression formation.

All of the face stimuli were created by the common procedure described above; however, they contained different images. We chose six pairs (two each for the three traits: competence, dominance, and trustworthiness) from set A and four pairs (two pairs each for trustworthy-untrustworthy and dominant-submissive) from set B. Altogether, four trustworthy-untrustworthy, four dominant-submissive, and two competent-incompetent pairs were presented to each participant. Examples of the stimuli are shown in Table 1. Using Adobe Photoshop software, the images were cropped in contour and superimposed on a uniform white background. These were printed on glossy paper side-by-side.

**TABLE 1:** Examples of the stimuli. Images in the upper and middle rows were used in Experiment 1a.

Trustworthiness		Dominance		Competence	
High	Low	High	Low	High	Low
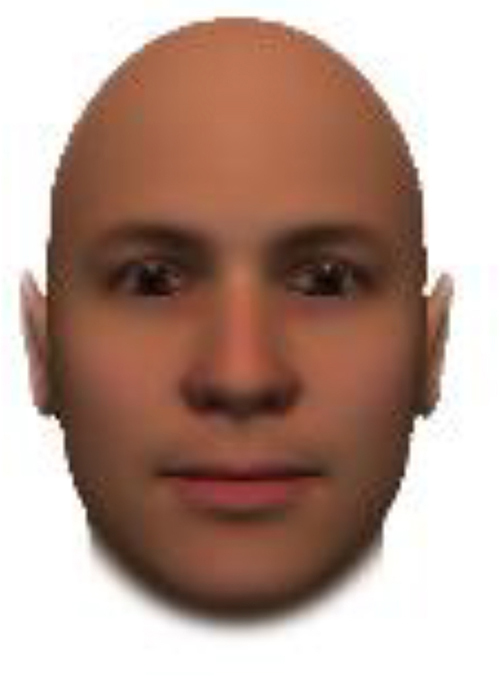	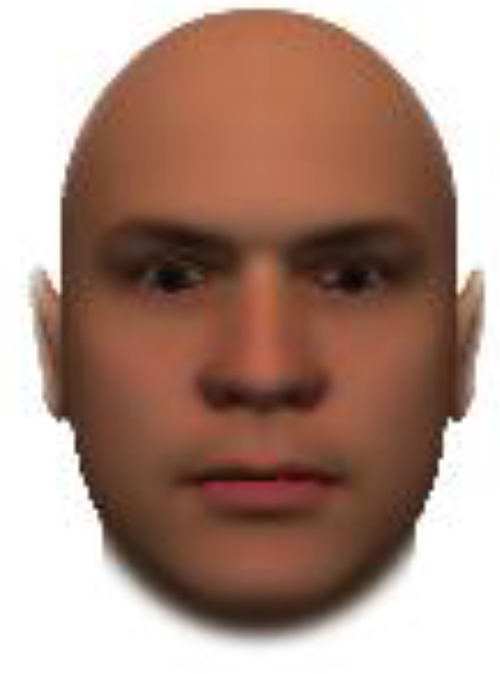	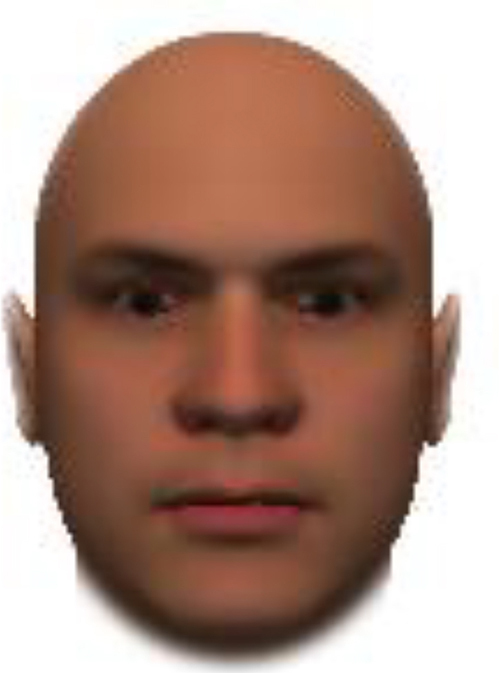	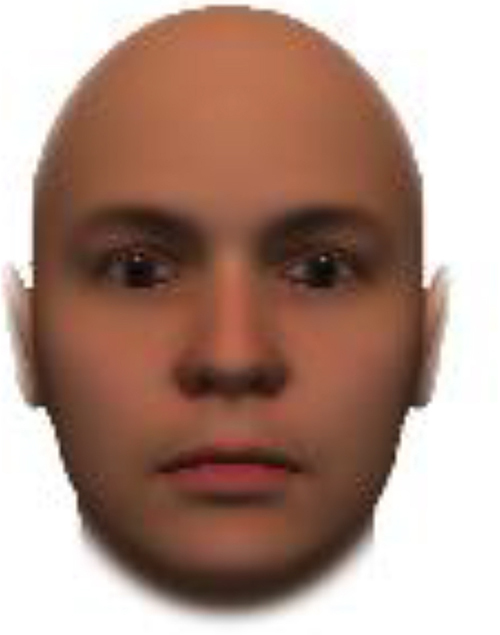		
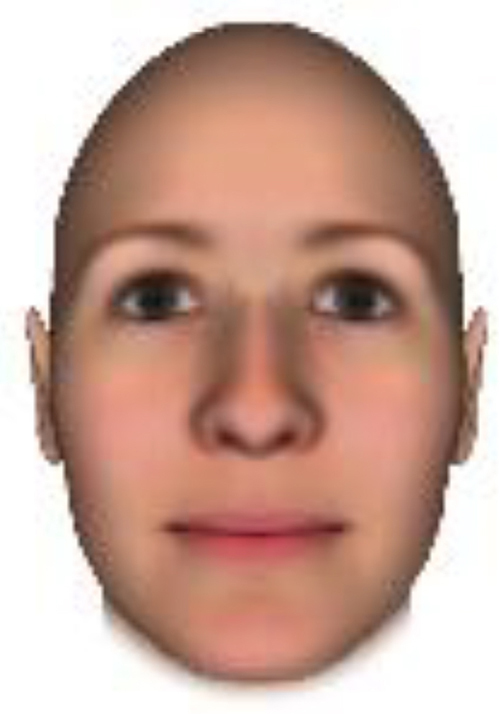	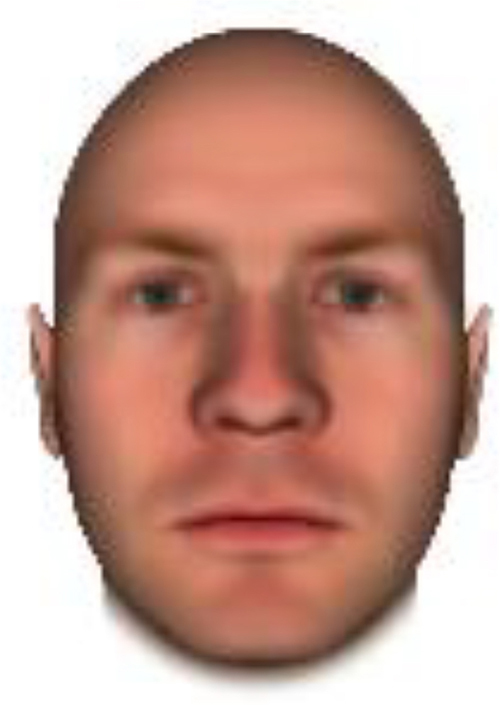	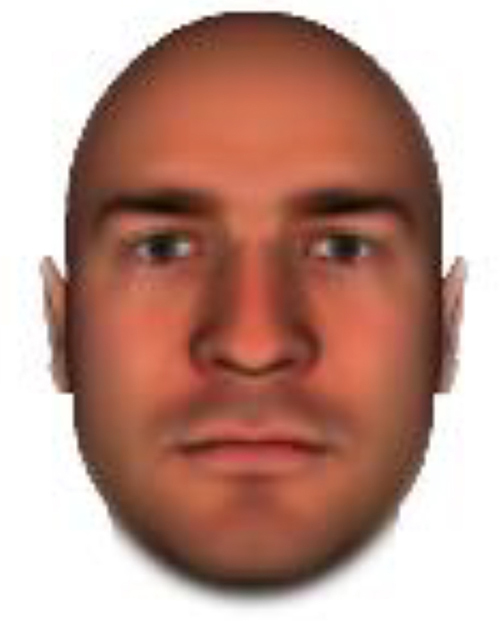	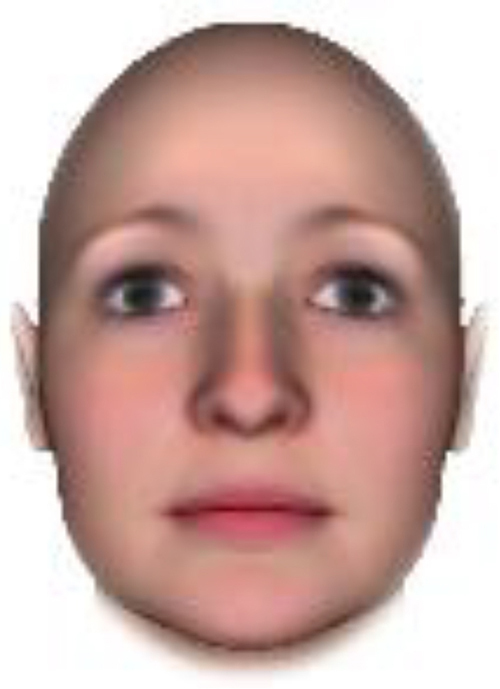	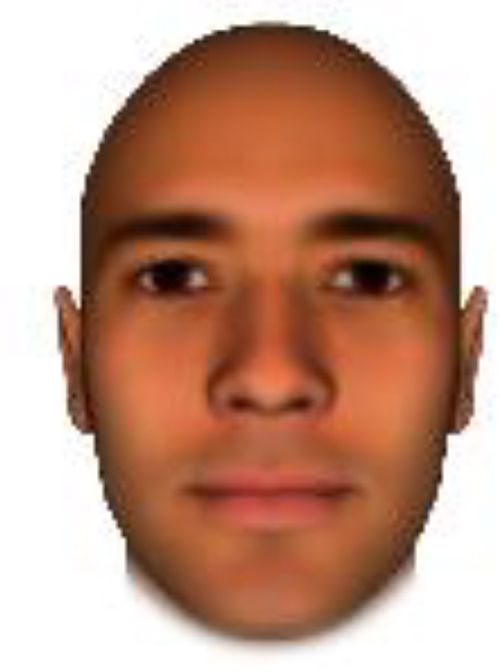	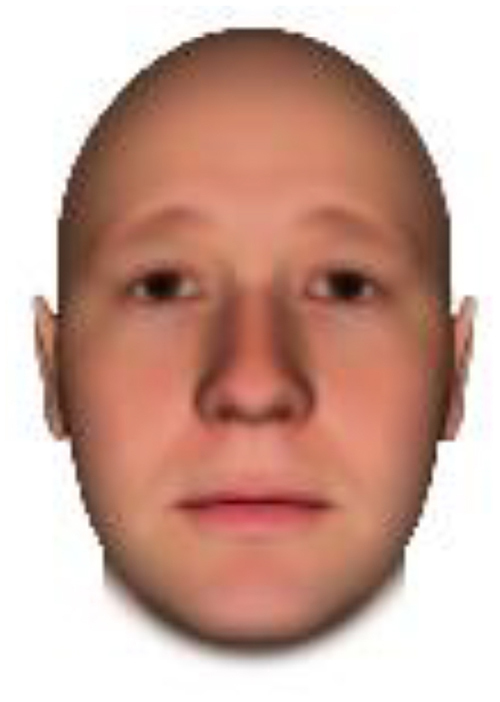
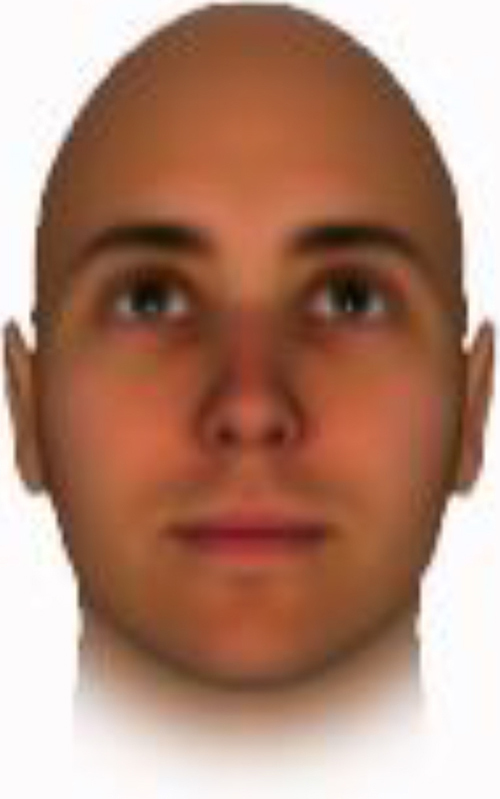	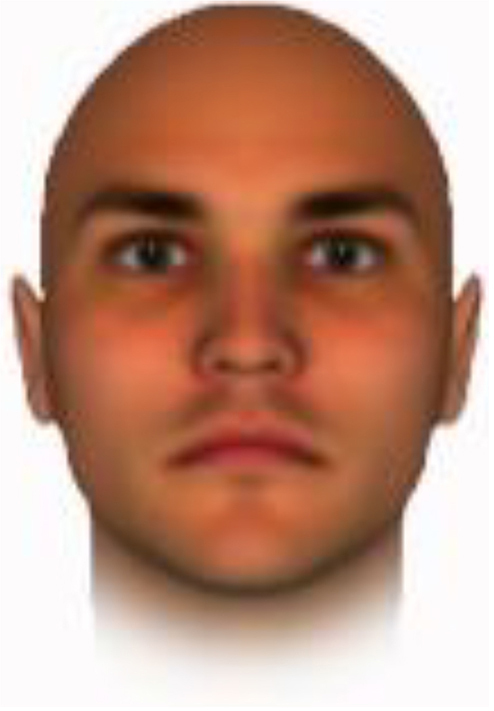	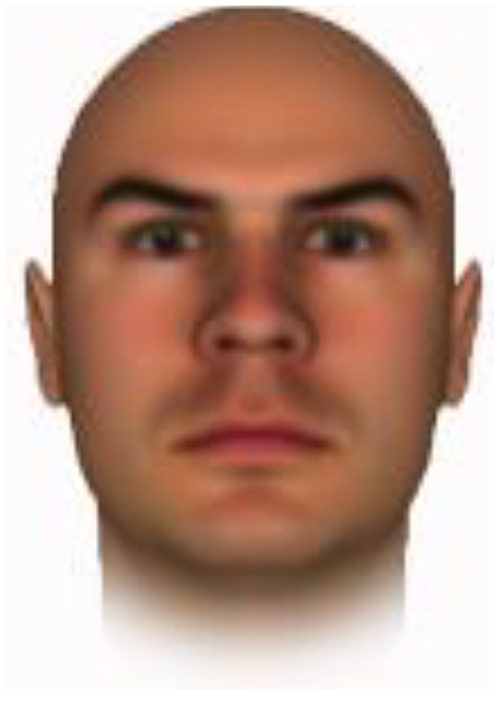	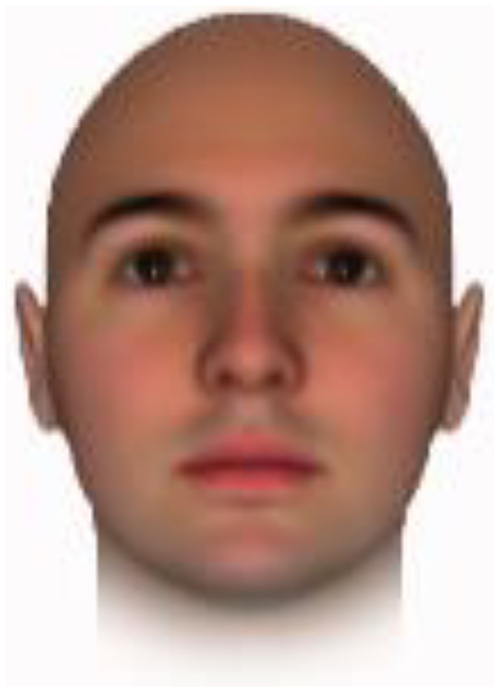	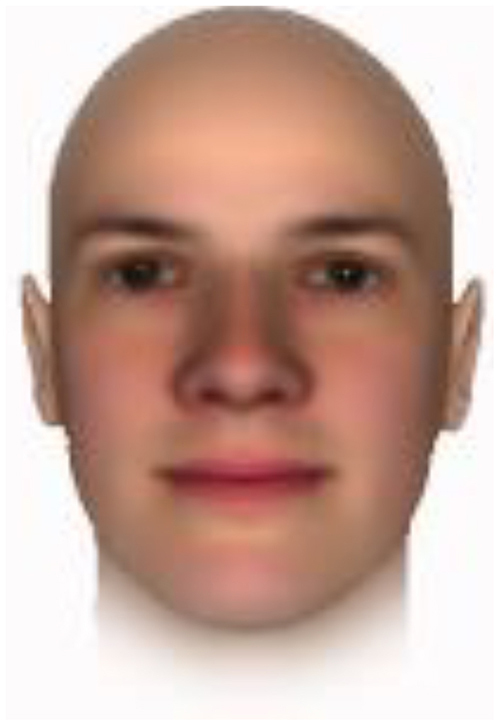	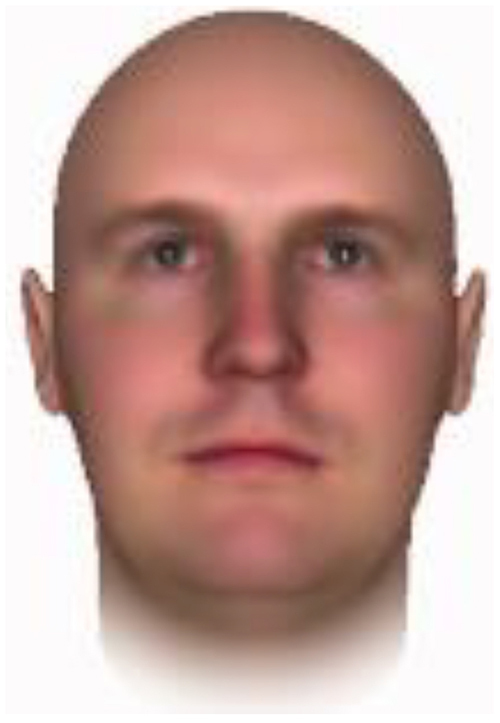

The upper four stimuli were identical to the stimuli used in Sakuta et al. (2018) and were taken from Oosterhof and Todorov (2008). Reproduced with permission. The middle six were chosen from Oosterhof and Todorov (2008). Reproduced with permission. Images in the lower row were added in Experiments 1b and 2. They were identical to the stimuli used in Cogsdill et al. (2014). (Source: https://osf.io/p85eb).

#### Procedure

Participants performed two-alternative forced-choice judgments in each pair. First, an experimenter showed two cartoon characters to relax and explain to the child what to do in the experiment, and confirmed that they were able to complete the task. Next, two characters were presented side-by-side on a white panel and the participants were asked to choose the one that they thought was better. Further, two stimuli were presented side-by-side as well. Participants were asked to judge which person was better (trustworthy), stronger (dominant), or smarter (competent). Each participant was exposed to 10 trials. Presentation order and position were counterbalanced across the participants. For example, a face that was judged as trustworthy by American adults (Oosterhof and Todorov, 2008) was presented on the left for half of the participants and on the right for the other half. Each participant’s behavior was recorded both in the video and recording sheets.

### Results

**FIGURE 1:**
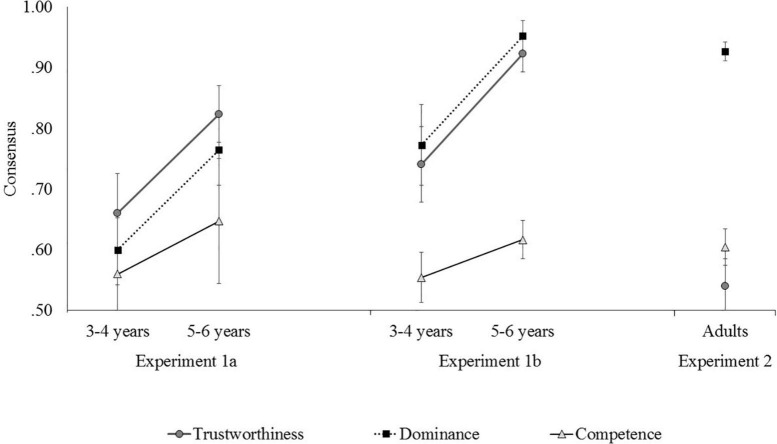
Consensus data for Japanese young children (Experiments 1a and 1b) and adults (Experiment 2) with the U.S. adults are shown together. (The error bar indicates standard error).

For each face in each stimulus pair, the percentage of children who selected the trustworthy face as the “nice” one was recorded for trustworthy-untrustworthy pair. Higher percentages indicated stronger consensus between Japanese children and American adults. Consensus data are shown in Figure 1 together with the data from Experiments 1b and 2. Please refer to the Supplementary material for the individual data. Frequencies of the choice were shown in Supplementary Table 1.

As a result of a one sample *t*-test against chance level, the difference was significant only for trustworthiness among the 3–4-year-olds [*t*(24) = 2.43, *p* = 0.02, *r* = 0.44], and significant for trustworthiness and dominance among the 5–6-year-olds [trustworthiness: *t*(16) = 6.91, *p* < 0.001, *r* = 0.87; dominance: *t*(16) = 4.52, *p* < 0.001, *r* = 0.75]. That is, both age groups showed significant consensus when identifying faces as mean or nice (66.00% for 3–4-year-olds; 82.35% for 5–6-year-olds). Only older children showed a significant consensus when identifying faces as strong or not strong (60.00% for 3–4-year-olds; 76.47% for 5–6-year-olds). No significant consensus above chance was shown for dominance among the 3–4-year-olds [60.00%, *t*(24) = 1.73, *p* = 0.10, *r* = 0.33] and smart or not smart pairs [56.00% for 3–4-year-olds, *t*(24) = 0.65, *p* = 0.52, *r* = 0.13; 64.71% for 5–6-year-olds, *t*(16) = 1.43, *p* = 0.17, *r* = 0.34].

A two-way analysis of variance (ANOVA) of age group × type of trait for the consensus rate revealed a significant main effect of age group, *F*(1, 40) = 5.94, *p* = 0.02, partial η^2^ = 0.13. Although the 3–4-year-olds responded with American adult like consensus, they were less consistent than the 5–6-year-olds. The main effect of trait and the interaction between age group and trait were not significant, *F*(2, 80) = 1.52, *p* = 0.23, partial η^2^ = 0.04; and *F*(2, 80) = 0.16, *p* = 0.82, partial η^2^ = 0.004, respectively.

### Discussion

Some differences were found in the development of impression formation. Based on the *t*-tests, for trustworthiness and dominance, older children showed better agreement with American adults than younger children. For competence, there was no relationship between ages and impression judgment. In sum, trustworthiness and dominance judgment became more consistent as children grew up, whereas competence judgment may have been more difficult for the children. However, as the ANOVA showed that only the main effect of age was significant and the interaction was not, it can be said that the overall agreement rate varies with age, although it cannot be said that the timing of development is different for the different types of impressions.

## Experiment 1b: Impression formation in Japanese children (Replication of Experiment 1a)

Experiment 1a used the same impression-manipulated stimulus set as Cogsdill et al. (2014); however, it was not identical. Therefore, in Experiment 1b, a stimulus set identical to that of Cogsdill et al. (2014) was added and the impression judgments from faces in Japanese children examined. If this experiment showed a tendency consistent with the results of Experiment 1a, more robust findings should be obtained on impression judgments in early childhood in Japan.

### Method

#### Participants

This study included 46 children (25 girls and 21 boys) divided into two age groups according to their class: 3–4-year-olds (*n* = 22) and 5–6-year-olds (*n* = 24). Although the year ranges were almost equivalent to Experiment 1a, 29 children did not disclose their birth month. The experiment was conducted from February to March 2018. There were eight children who were categorized into younger group in Experiment 1a and in the older group in Experiment 1b. All the children were Japanese and recruited from a nursery school. Sixteen children had also participated in Experiment 1a. The experiments were conducted according to the principles laid down in the Declaration of Helsinki. Written informed consent was obtained from each child’s parents prior to their participation.

#### Stimuli

The same 10 stimuli pairs as in Experiment 1a were used again. Additionally, 18 stimuli were chosen from Cogsdill et al. (2014). Altogether seven trustworthy-untrustworthy, seven dominant-submissive, and five competent-incompetent pairs were used. Using Adobe Photoshop software, the images were cropped in contour and superimposed on a uniform white background. These were printed on glossy paper side-by-side.

#### Procedure

The procedure was identical to that of Experiment 1a. Each participant’s behavior was recorded both in the video and recording sheets.

### Results

As per Experiment 1a, the percentages of consensus were calculated (Figure 1). Frequencies of the choice were shown in Supplementary Table 2. As a result of a one sample *t*-test versus chance level, both age groups showed significant consensus when identifying faces as mean or nice [74.03% for 3–4-year-olds, *t*(21) = 3.87, *p* < 0.001, *r* = 0.65; 92.26% for 5–6-year-olds, *t*(23) = 14.20, *p* < 0.001, *r* = 0.95] and as strong or weak [77.27% for 3–4-year-olds, *t*(21) = 4.08, *p* < 0.001, *r* = 0.67; 95.24% for 5–6-year-olds, *t*(23) = 17.87, *p* < 0.001, *r* = 0.97]. Only older children showed significant consensus when identifying faces as smart or not smart [55.45% for 3–4-year-olds, *t*(21) = 1.32, *p* = 0.20, *r* = 0.28; 61.67% for 5–6-year-olds, *t*(23) = 3.69, *p* < 0.01, *r* = 0.61].

A two-way ANOVA revealed significant main effects of age group and trait, *F*(1, 44) = 12.14, *p* < 0.001, partial η^2^ = 0.22; *F*(2, 88) = 26.24, *p* < 0.001, partial η^2^ = 0.37, respectively. Although the 3–4-year-olds responded with adult like consensus, they were less consistent than the 5–6-year-olds. Competence judgment showed lower consensus than trustworthiness and dominance. The interaction between age group and trait was not significant, *F*(2, 88) = 1.34, *p* = 0.27, partial η^2^ = 0.03.

### Discussion

We found some differences in the development of impression formation, as in Experiment 1a. Compared to Experiment 1a, the overall consensus in judgment with American adults increased; however, the overall trend was similar to that of Experiment 1a. For all impressions, older children showed better agreement with American adults than younger children. Trustworthiness and dominance judgment became more consistent as children grew up, whereas competence judgment may have been more difficult for the children. However, as in Experiment 1a, the interaction between age group and trait was not significant. Therefore, it cannot be said that the rate of consensus in trait judgments differs by age.

## Experiment 2: Impression formation in Japanese adults

Using the identical stimuli set, Japanese adults’ impression judgment was examined and compared to that of Japanese children (data from Experiments 1a and 1b) and American adults (Cogsdill et al., 2014).

### Method

#### Participants

This study included 45 Japanese adults (33 women and 12 men, mean age: 20.3 years old, range: 18–23 years old). Participants provided informed consent to the procedures, and the study was approved by the Ethical Committee of Jissen Women’s University. The experiment was hosted online using a Google form and participants were recruited through the University. The experiments were conducted according to the principles laid down in the Declaration of Helsinki.

#### Stimuli

We used identical stimulus sets to Experiment 1b. Altogether, seven trustworthy-untrustworthy, seven dominant-submissive, and five competent-incompetent pairs were used in Experiment 2.

#### Procedure

Participants were asked to judge which person was better (trustworthy), stronger (dominant), or smarter (competent). Different from Experiments 1a and 1b, the adults performed a forced-choice judgment with 5-point confidence rating scales and answered using an online form (Google form). For example, to the question “Which person seems to be more trustworthy?” participants were required to choose one of the following: 1: definitely left, 2: probably left, 3: not sure, 4: probably right, 5: definitely right. Presentation order and position were counterbalanced among the participants.

### Results

The percentages of responses were calculated along with the five response categories for each stimulus pair. The five categories indicated the five degrees varied from the unexpected response with higher confidence (i.e., low consensus with the American judgment) to expected response with higher confidence (i.e., high consensus with the American judgment). Thus, they corresponded with the degree of consensus to the American adults’ judgment. As a result of a chi-squared test, all traits showed significance [trustworthiness: χ^2^(4) = 49.33, *p* < 0.01, dominance: χ^2^(4) = 442.03, *p* < 0.01, and competence: χ^2^(4) = 62.80, *p* < 0.01]. As shown in Table 2, dominance judgment showed the highest consensus.

**TABLE 2:** Japanese adults’ impression judgment (choice rate; %) and the result of the chi-squared-test in Experiment 2.

	Consensus with U.S.					
	Low			High		
	Very confident (definitely)	Less confident (probably)	Not sure	Less confident (probably)	Very confident (definitely)	*χ2*
Trustworthiness	6.98	20.00	19.05	31.75	22.22	49.33, *p* < 0.01
Dominance	1.59	0.95	4.76	30.79	61.90	442.03, *p* < 0.01
Competence	7.11	13.78	18.67	38.67	21.78	62.80, *p* < 0.01

χ2 indicates the chi-squared test. Choice rate for each of the five categories was calculated for three types of stimuli sets. For example, when a participant chose a left image as trustworthy with high confidence, and the image was a trustworthy face in the U.S., it was qualified as “high consensus with U.S”.

Next, we summed the selection frequencies of “definitely left” and “probably left” and “probably right” and “definitely right” for each stimulus pair to create judgment categories consistent and inconsistent with the American judgments, respectively. The percentage of responses for each judgment categories was calculated, and a two-way ANOVA with age group and type of trait as factors was performed. Participants’ data from Experiments 1a and 1b were also used for this analysis. Averages of agreement with U.S. were shown in Supplementary Table 3. As noted above, 16 children participated in both experiments; in Experiment 2, these participants’ data were regarded as independent from that of the participants who were different in both experiments. All main effects and an interaction were significant, age group: *F*(2, 130) = 10.46, *p* < 0.001, partial η^2^ = 0.14; trait: *F*(2, 260) = 25.40, *p* < 0.001, partial η^2^ = 0.16; interaction: *F*(4, 260) = 9.53, *p* < 0.001, partial η^2^ = 0.13. Simple main effect tests for interaction showed that differences among traits were significant in all age groups (3–4-year-olds: *p* = 0.02, 5–6-year-olds: *p* < 0.001, adults: *p* < 0.001). Multiple comparisons using Holm’s method showed that dominance judgment (92.7%) was significantly more consistent than trustworthiness (54.0%) and competence (60.4%) judgments in adults (adjusted *p*s < 0.01). Competence judgment (62.9%) was less consistent than the other two traits (trustworthiness: 88.2%, dominance: 87.5%) in 5–6-year-olds (adjusted *p*s < 0.01). This tendency was also seen in 3–4-year-olds, but the adjusted *p*-value was not significant (trustworthiness: 69.8%, dominance: 68.1%, competence: 55.7%). In terms of traits, the difference in consensus rates among age groups were significant for trustworthiness (*p* < 0.001) and dominance (*p* < 0.001), but not for competence (*p* = 0.43). Adults had the lowest consensus rate for trustworthiness judgments (54.0%), followed by 3–4-year-olds (69.8%) and 5–6-year-olds (88.2%). For dominance judgments, adults (92.7%) and 5–6-year-olds (87.5%) had similar consensus rates, with 3–4-year-olds having the lowest rates (68.1%).

### Discussion

When data obtained from Japanese children and adults were analyzed together, it was found that the consensus with the U.S. data increased with age for the dominance judgment. However, the consensus for trustworthiness judgment decreased to near chance level in adults compared to children. Competence judgment was approximately 60% consensus in all age groups and did not vary with age. The results showed that some impression judgments became more similar between Japanese and American judgments as the respondents’ age increased, while others decreased in consensus. Trustworthiness was more likely to be influenced by culture and experience, as consensus was lower in adults compared to early childhood.

## General discussion

Overall, in Experiment 1a, agreement with the American judgments increased from ages 3–4 to 5–6 years. Competence judgments showed a relatively low agreement with Americans (56–64%). These results were consistent with the prediction that trustworthiness and dominance would be more universal judgment dimensions, and competence would be more influenced by culture and experience. Using the same stimulus set, Sakuta et al. (2018) found that for trustworthiness, Japanese infants also showed preferential gazing at face images that Americans judged trustworthy. Therefore, Japanese infants tended to agree with the American judgments regarding trustworthiness. In addition, Cogsdill et al. (2014) reported that American children judged competence similarly to American adults, with agreement rates of 75% at ages 3–4 and nearly 90% at ages 5–6 years old. This suggested that the low agreement rate in competence judgments in Experiment 1a was not solely due to the underdevelopment of the judgment criteria in early childhood. It was also likely influenced by culture. Experiment 1b was conducted with the same additional stimulus set as in Cogsdill et al. (2014). Overall, agreement with Cogsdill et al. (2014) increased; however, the trend was the same as in Experiment 1a. Trustworthiness and dominance had higher agreement rates, while competence had lower agreement rates. The overall increase in agreement was due to the addition of the same stimulus set as in the previous study. Hence, this was not surprising. However, it is possible that the stimuli judged to be highly trustworthy in the U.S. may also be judged to be highly trustworthy or not in Japan, depending on the images used.

As 16 of the 46 participants in Experiment 1b also participated in Experiment 1a, the possibility that these children’s experience with inferring traits from faces in Experiment 1a may have influenced their face-to-trait inferences in Experiment 1b cannot be ruled out. However, in this study, participants were not given individual feedback regarding the results of their judgments. Therefore, it was impossible for them to obtain information such as how the face image they selected was judged in the U.S. (e.g., whether the person was considered more trustworthy). Therefore, it is unlikely that children who participated in both experiments would have a higher rate of agreement with the American participants than children who participated only once. Furthermore, the mean agreement rates for children who participated only in Experiment 1b (*n* = 30) and those who participated in both (*n* = 16) were found to be 77.3 and 74.4%, respectively, and a *t*-test showed no significant difference [*t*(44) = 0.61, *p* = 0.55]. In summary, while it is conceivable that past experience with trait inference may influence subsequent trait inferences, it does not appear to be a reason for the higher agreement rate in Experiment 1b than in Experiment 1a in the present data.

Experiment 2 used the same stimulus set as Experiment 1b and was conducted with Japanese adults. Only dominance was in agreement with Cogsdill et al. (2014) at over 90%, whereas trustworthiness and competence had only 54 and 60% judgmental agreement, respectively. These results were consistent with the prediction that competence would be influenced by culture, yet contrary to the prediction that trustworthiness would be a universal dimension. In particular, the finding that trustworthiness judgments were less consistent with American judgments in adults than in infancy was surprising.

As for the difference in trustworthiness judgment, it is possible that there is a difference in the meaning of a smile between Japan and America. Regarding evaluation, smiling has generally been thought to be positively valued. However, when Krys et al. (2014) compared intellectual impressions of smiling people across seven countries, they found that in Germany and China smiling people were perceived as more intelligent than non-smiling people, while in Iran, on the contrary, they were perceived as less intelligent. It was shown that in some cultures, smiling people may be evaluated more negatively than non-smiling people. Matsumoto and Kudoh (1993) also reported the differences of trait judgment on smiling and non-smiling faces between American and Japanese raters. For example, Americans rated a smiling person as more intelligent than a neutral face, while the Japanese did not show such a difference. Furthermore, while there was agreement that smiling was more sociable than a neutral face, the degree of sociability was greater among Americans than among the Japanese. These findings suggest that due to cultural differences in facial expression recognition, there is a strong possibility that cultural differences also occur in impression perception, considered to be dependent on facial expressions.

There are two possible factors, other than cultural differences in the evaluations themselves, that may explain the present results: the race of the face and terms of the evaluation.

First, it should be noted that this may include the influence of other-race effects. The other-race effect is a phenomenon in which the face of another race is more difficult to distinguish than the face of one’s own race, and it has been observed even in infants less than 1 year old (Kelly et al., 2009, 2007). As composite images based on Caucasian faces were used for Japanese children and adults in this study, the possibility that the obtained results included only differences in impression perception due to other-race effects and culture cannot be denied. Among the results obtained in this study, the other-race effect was possibly involved in the fact that the agreement rate with the judgment of Americans was lower for adults than for infants. However, Japanese infants aged 6–8 months old have been found to gaze at faces judged trustworthy by American adults in experiments with Caucasian faces (Sakuta et al., 2018). Thus, at the very least, infants were able to detect some physical cues that induce the impression of trustworthiness, regardless of the race of the face. However, as described in the Introduction, it cannot be said that they merely gazed at faces that appeared to smile. Therefore, it may be necessary to include cues other than facial expressions in regard to infants’ impression perception. As, currently, very few studies on cultural differences in impression perception from faces have been conducted, further research is needed.

Second, when examining cultural differences, it is necessary to consider the differences in word connotations. In Cogsdill et al. (2014), trustworthiness was translated as “mean/nice,” dominance as “strong/not strong,” and competence as “smart/not smart.” It may be debatable whether this paraphrasing was appropriate, that is, whether trustworthy had exactly the same meaning as nice for adults. In addition, it is possible that the words “trustworthiness,” “dominance,” and “competence” in the U.S. and “*shinraikan*,” “*shihaisei*,” and “*yuunousa*” in Japan, as well as “nice,” “strong,” and “smart,” paraphrases of these words for young children in the U.S., and “*iihitosou*,” “*tsuyosou*,” and “*atama ga yosasou*” for young children in Japan do not necessarily have the exact same meanings. It is also possible that the meaning of “trustworthy” as used by adults is different between the U.S. and Japan. The English word “nice” and the Japanese word “*ii* (良い)” are thought to correspond almost semantically. However, the Japanese word “*shinraidekiru* (信頼できる＝trustworthy)” does not necessarily mean the same thing as a “good” person. We cannot deny the possibility that the intentional impression-judgment task and verbal response may have resulted in subtle differences in nuance, or that the instruction was not well conveyed to younger children. Therefore, in the future, it will be necessary to examine cultural differences in impression perception without language by conducting cognitive tasks that do not depend on language.

Recently, the Western, Educated, Industrialized, Rich, and Democratic (WEIRD) problem has been noted (Henrich et al., 2010). Namely, many articles regarding psychology have been published based on samples drawn entirely from WEIRD societies. Furthermore, in social cognition, many studies are still published from the U.S., Europe, and other countries, and the findings may be biased as mainly Caucasian faces are used (Cook and Over, 2021). The current study examined the impressions that Japanese children and adults had of computer-generated images based on Caucasian faces. By using the same images as in a previous study conducted in the U.S., the responses to the same stimuli could be directly compared. This is a big advantage in examining the basic face perception. In the future, it may be possible to examine the generalizability of the findings by mixing the faces of various races and using realistic human face images. In this study, CG composite images were used; however, realistic facial images can be used to reproduce realistic situations and provide findings with greater ecological validity.

## Conclusion

The findings of this study can be summarized in two points: First, while the trend in Japanese young children becomes more similar to that in Americans as they get older, the degree of agreement in the judgment of trustworthiness decreases in adults, suggesting that the criteria for judging impressions from faces changes significantly between early childhood and adulthood. Second, the results suggest that some criteria for such impression judgments are less susceptible to cultural influences than others, depending on the dimension of the trait. In this study, contrary to expectations, it was the trustworthiness impression that was more susceptible to the influence of culture. Together with the adults’ data, there could be some differences among cultures and ages. By approaching the cultural differences in impression perception from the aspect of impression perception in early childhood, the differences among impression dimensions were examined in detail. Recognizing that there can be discrepancies in cross-cultural communication due to differences in impression judgments and facial expression recognition from the face by accumulating such studies will be useful for forming smooth cross-cultural communication.

## Data availability statement

The original contributions presented in this study are included in the article/Supplementary material, further inquiries can be directed to the corresponding author.

## Ethics statement

The studies involving human participants were reviewed and approved by the Ethics Committee of Jissen Women’s University. Written informed consent to participate in this study was provided by the participants or their legal guardian/next of kin in Experiments 1a and 1b and from the participants themselves in Experiment 2.

## Author contributions

YS designed the study, conducted the experiments, analyzed the results, and wrote the manuscript.

## Footnotes

1
http://www.facegen.com/

2
https://tlab.uchicago.edu/databases/

## References

[B1] Antonakis J. Dalgas O. (2009). Predicting elections: Child’s play! *Science* 323:1183. 10.1126/science.1167748 19251621

[B2] Blais C. Jack R. E. Scheepers C. Fiset D. Caldara R. (2008). Culture shapes how we look at faces. *PLoS One* 3:e3022. 10.1371/journal.pone.0003022 18714387 PMC2515341

[B3] Blanz V. Vetter T. (1999). “A morphable model for the synthesis of 3D faces,” in *Proceedings of the 26th Annual Conference on Computer Graphics and Interactive Techniques*, (New York, NY: SIGGRAPH), 187–194. 10.1145/311535.311556

[B4] Brace N. A. Hole G. J. Kemp R. I. Pike G. E. Van Duuren M. Norgate L. (2001). Developmental changes in the effect of inversion: Using a picture book to investigate face recognition. *Perception* 30 85–94. 10.1068/p3059 11257980

[B5] Cogsdill E. J. Banaji M. R. (2015). Face-trait inferences show robust child-adult agreement: Evidence from three types of faces. *J. Exp. Soc. Psychol.* 60 150–156. 10.1016/j.jesp.2015.05.007

[B6] Cogsdill E. J. Todorov A. T. Spelke E. S. Banaji M. R. (2014). Inferring character from faces: A developmental study. *Psychol. Sci.* 25 1132–1139. 10.1177/0956797614523297 24570261 PMC5580985

[B7] Cook R. Over H. (2021). Why is the literature on first impressions so focused on white faces? *R. Soc. Open Sci.* 8:211146. 10.1098/rsos.211146 34567592 PMC8456137

[B8] Cuddy A. J. C. Fiske S. T. Glick P. (2008). Warmth and competence as universal dimensions of social perception: The stereotype content model and the BIAS map. *Adv. Exp. Soc. Psychol.* 40 61–149. 10.1016/S0065-2601(07)00002-0

[B9] Cunningham M. R. Roberts A. R. Barbee A. P. Druen P. B. Wu C.-H. (1995). ‘Their ideas of beauty are, on the whole, the same as ours’: Consistency and variability in the cross-cultural perception of female physical attractiveness. *J. Pers. Soc. Psychol.* 68 261–279. 10.1037/0022-3514.68.2.261

[B10] Ewing L. Sutherland C. A. M. Willis M. L. (2019). Children show adult-like facial appearance biases when trusting others. *Dev. Psychol.* 55 1694–1701. 10.1037/dev0000747 31045400

[B11] Fiske S. T. Cuddy A. J. C. Glick P. (2007). Universal dimensions of social cognition: Warmth and competence. *Trends Cogn. Sci.* 11 77–83. 10.1016/j.tics.2006.11.005 17188552

[B12] Henrich J. Heine S. J. Norenzayan A. (2010). The weirdest people in the world? *Behav. Brain Sci* 33 61–83. 10.1017/S0140525X0999152X 20550733

[B13] Ichikawa H. Kanazawa S. Yamaguchi M. K. (2014). Infants recognize the subtle happiness expression. *Perception* 43 235–248. 10.1068/p7595 25109015

[B14] Jack R. E. Blais C. Scheepers C. Schyns P. G. Caldara R. (2009). Cultural confusions show that facial expressions are not universal. *Curr. Biol.* 19 1543–1548. 10.1016/j.cub.2009.07.051 19682907

[B15] Jessen S. Grossmann T. (2016). Neural and behavioral evidence for infants’ sensitivity to the trustworthiness of faces. *J. Cogn. Neurosci.* 28 1728–1736. 10.1162/jocn_a_00999 27315276

[B16] Jessen S. Grossmann T. (2019). Neural evidence for the subliminal processing of facial trustworthiness in infancy. *Neuropsychologia* 126 46–53. 10.1016/j.neuropsychologia.2017.04.025 28442339

[B17] Kelly D. J. Liu S. Lee K. Quinn P. C. Pascalis O. Slater A. M. (2009). Development of the other-race effect during infancy: Evidence toward universality? *J. Exp. Child Psychol.* 104 105–114. 10.1016/j.jecp.2009.01.006 19269649 PMC3740564

[B18] Kelly D. J. Quinn P. C. Slater A. M. Lee K. Ge L. Pascalis O. (2007). The other-race effect develops during infancy: Evidence of perceptual narrowing. *Psychol. Sci.* 18 1084–1089. 10.1111/j.1467-9280.2007.02029.x 18031416 PMC2566514

[B19] Krys K. Hansen K. Xing C. Szarota P. Yang M. (2014). Do only fools smile at strangers? cultural differences in social perception of intelligence of smiling individuals. *J. Cross Cult. Psychol.* 45 314–321. 10.1177/0022022113513922

[B20] Lee R. Flavell J. C. Tipper S. P. Cook R. Over H. (2021). Spontaneous first impressions emerge from brief training. *Sci. Rep.* 11:15024. 10.1038/s41598-021-94670-y 34294809 PMC8298428

[B21] Matsumoto D. Kudoh T. (1993). American-Japanese cultural differences in attributions of personality based on smiles. *J. Nonverbal Behav.* 17 231–243. 10.1007/BF00987239

[B22] Mondloch C. J. Gerada A. Proietti V. Nelson N. L. (2019). The influence of subtle facial expressions on children’s first impressions of trustworthiness and dominance is not adult-like. *J. Exp. Child Psychol.* 180 19–38. 10.1016/j.jecp.2018.12.002 30611111

[B23] Oosterhof N. N. Todorov A. (2008). The functional basis of face evaluation. *Proc. Natl Acad. Sci. U.S.A.* 105 11087–11092. 10.1073/pnas.0805664105 18685089 PMC2516255

[B24] Osgood C. E. Suci G. J. Tannenbaum P. H. (1957). *The Measurement of Meaning.* Champaign, IL: University of Illinois Press.

[B25] Pascalis O. Scott L. S. Kelly D. J. Shannon R. W. Nicholson E. Coleman M. (2005). Plasticity of face processing in infancy. *Proc. Natl. Acad. Sci. U.S.A.* 102 5297–5300. 10.1073/pnas.0406627102 15790676 PMC555965

[B26] Rule N. O. Ambady N. Adams R. B. Ozono H. Nakashima S. Yoshikawa S. (2010). Polling the face: Prediction and consensus across cultures. *J. Pers. Soc. Psychol.* 98 1–15. 10.1037/a0017673 20053027

[B27] Rule N. O. Ishii K. Ambady N. (2011). Cross-cultural impressions of leaders’ faces: Consensus and predictive validity. *Int. J. Intercult. Relat.* 35 833–841. 10.1016/j.ijintrel.2011.06.001

[B28] Sakuta Y. Ishi H. Akamatsu S. Gyoba J. (2009). Psychological evaluation of higher-order facial impressions synthesized by the impression transfer vector method. *Kansei Eng. Int. J.* 9 1–10. 10.5057/kei.9.1

[B29] Sakuta Y. Kanazawa S. Yamaguchi M. K. (2018). Infants prefer a trustworthy person: An early sign of social cognition in infants. *PLoS One* 13:e0203541. 10.1371/journal.pone.0203541 30188941 PMC6126855

[B30] Sutherland C. A. M. Liu X. Zhang L. Chu Y. Oldmeadow J. A. Young A. W. (2018). Facial first impressions across culture: Data-driven modeling of Chinese and British perceivers’ unconstrained facial impressions. *Pers. Soc. Psychol. Bull.* 44 521–537. 10.1177/0146167217744194 29226785

[B31] Sutherland C. A. M. Oldmeadow J. A. Santos I. M. Towler J. Michael Burt D. Young A. W. (2013). Social inferences from faces: Ambient images generate a three-dimensional model. *Cognition* 127 105–118. 10.1016/j.cognition.2012.12.001 23376296

[B32] Sutherland C. A. M. Young A. W. Rhodes G. (2017). Facial first impressions from another angle: How social judgements are influenced by changeable and invariant facial properties. *Br. J. Psychol.* 108 397–415. 10.1111/bjop.12206 27443971

[B33] Tang Y. Harris P. L. Zou H. Xu Q. (2019). The impact of emotional expressions on children’s trust judgments. *Cogn. Emot.* 33 318–331. 10.1080/02699931.2018.1449735 29540092

[B34] Todorov A. (2008). Evaluating faces on trustworthiness: An extension of systems for recognition of emotions signaling approach/avoidance behaviors. *Ann. N. Y. Acad. Sci.* 1124 208–224. 10.1196/annals.1440.012 18400932

[B35] Todorov A. Oosterhof N. (2011). Modeling social perception of faces [Social Sciences]. *IEEE Signal Process. Mag.* 28 117–122. 10.1109/MSP.2010.940006

[B36] Todorov A. Dotsch R. Porter J. M. Oosterhof N. N. Falvello V. B. (2013). Validation of data-driven computational models of social perception of faces. *Emotion* 13 724–738. 10.1037/a0032335 23627724

[B37] Todorov A. Mandisodza A. N. Goren A. Hall C. C. (2005). Inferences of competence from faces predict election outcomes. *Science* 308 1623–1626. 10.1126/science.1110589 15947187

[B38] Todorov A. Pakrashi M. Oosterhof N. N. (2009). Evaluating faces on trustworthiness after minimal time exposure. *Soc. Cogn.* 27 813–833. 10.1521/soco.2009.27.6.813

[B39] Todorov A. Said C. P. Engell A. D. Oosterhof N. N. (2008). Understanding evaluation of faces on social dimensions. *Trends Cogn. Sci.* 12 455–460. 10.1016/j.tics.2008.10.001 18951830

[B40] van’t Wout M. Sanfey A. G. (2008). Friend or foe: The effect of implicit trustworthiness judgments in social decision-making. *Cognition* 108 796–803. 10.1016/j.cognition.2008.07.002 18721917

[B41] Van Der Zant T. Reid J. Mondloch C. J. Nelson N. L. (2021). The influence of postural emotion cues on implicit trait judgements. *Motiv. Emot.* 45 641–648. 10.1007/s11031-021-09889-z

[B42] Verosky S. C. Todorov A. (2013). When physical similarity matters: Mechanisms underlying affective learning generalization to the evaluation of novel faces. *J. Exp. Soc. Psychol.* 49 661–669. 10.1016/j.jesp.2013.02.004

[B43] Walker M. Jiang F. Vetter T. Sczesny S. (2011). Universals and cultural differences in forming personality trait judgments from faces. *Soc. Psychol. Pers. Sci.* 2 609–617. 10.1177/1948550611402519

[B44] Willis J. Todorov A. (2006). First impressions: Making up your mind after a 100-ms exposure to a face. *Psychol. Sci.* 17 592–598. 10.1111/j.1467-9280.2006.01750.x 16866745

[B45] Zebrowitz L. A. (2017). First impressions from faces. *Curr. Dir. Psychol. Sci.* 26 237–242. 10.1177/0963721416683996 28630532 PMC5473630

[B46] Zebrowitz L. A. Montepare J. M. Lee H. K. (1993). They don’t all look alike: Individual impressions of other racial groups. *J. Pers. Soc. Psychol.* 65 85–101. 10.1037//0022-3514.65.1.85 8355144

[B47] Zebrowitz L. A. Wang R. Bronstad P. M. Eisenberg D. Undurraga E. Reyes-García V. (2012). First impressions from faces among U.S. and culturally isolated Tsimane’ people in the Bolivian rainforest. *J. Cross Cult. Psychol.* 43 119–134. 10.1177/0022022111411386

