# Supplementary material for: Face-to-trait inferences in Japanese children and adults based on Caucasian faces
Source: Front Psychol. 2022 Dec 7;13:955194. doi: 10.3389/fpsyg.2022.955194 (PMC9769403; doi:10.3389/fpsyg.2022.955194)
Supplement: Supplementary file 1 [file Data_Sheet_1.docx]

Supplementary Material

# Supplementary Data

Agreement (%) with U.S. in Experiment 1a.

| **ID** | **Gender** | **Age group** | **Trustworthiness** | **Dominance** | **Competence** |
| --- | --- | --- | --- | --- | --- |
| **1** | Female | 3-4yo | 25.0 | 50.0 | 100.0 |
| **2** | Female | 3-4yo | 75.0 | 25.0 | 100.0 |
| **3** | Female | 3-4yo | 75.0 | 0.0 | 50.0 |
| **4** | Female | 3-4yo | 50.0 | 75.0 | 0.0 |
| **5** | Male | 3-4yo | 50.0 | 25.0 | 100.0 |
| **6** | Female | 3-4yo | 75.0 | 25.0 | 100.0 |
| **7** | Female | 3-4yo | 100.0 | 50.0 | 50.0 |
| **8** | Male | 3-4yo | 75.0 | 100.0 | 100.0 |
| **9** | Female | 3-4yo | 75.0 | 75.0 | 0.0 |
| **10** | Female | 3-4yo | 100.0 | 50.0 | 0.0 |
| **11** | Male | 3-4yo | 100.0 | 100.0 | 0.0 |
| **12** | Male | 3-4yo | 100.0 | 50.0 | 100.0 |
| **13** | Female | 3-4yo | 0.0 | 75.0 | 100.0 |
| **14** | Female | 3-4yo | 75.0 | 50.0 | 100.0 |
| **15** | Female | 3-4yo | 75.0 | 75.0 | 50.0 |
| **16** | Female | 3-4yo | 100.0 | 100.0 | 0.0 |
| **17** | Female | 3-4yo | 75.0 | 0.0 | 0.0 |
| **18** | Male | 3-4yo | 75.0 | 100.0 | 100.0 |
| **19** | Male | 3-4yo | 0.0 | 75.0 | 100.0 |
| **20** | Female | 3-4yo | 25.0 | 50.0 | 100.0 |
| **21** | Female | 3-4yo | 50.0 | 75.0 | 0.0 |
| **22** | Female | 3-4yo | 100.0 | 75.0 | 50.0 |
| **23** | Female | 3-4yo | 100.0 | 75.0 | 0.0 |
| **24** | Male | 3-4yo | 0.0 | 50.0 | 0.0 |
| **25** | Male | 3-4yo | 75.0 | 75.0 | 100.0 |
| **26** | Female | 5-6yo | 75.0 | 100.0 | 100.0 |
| **27** | Male | 5-6yo | 75.0 | 25.0 | 50.0 |
| **28** | Female | 5-6yo | 50.0 | 100.0 | 100.0 |
| **29** | Male | 5-6yo | 100.0 | 50.0 | 0.0 |
| **30** | Male | 5-6yo | 100.0 | 100.0 | 50.0 |
| **31** | Male | 5-6yo | 100.0 | 50.0 | 50.0 |
| **32** | Male | 5-6yo | 75.0 | 75.0 | 100.0 |
| **33** | Female | 5-6yo | 100.0 | 100.0 | 100.0 |
| **34** | Male | 5-6yo | 50.0 | 75.0 | 0.0 |
| **35** | Female | 5-6yo | 50.0 | 50.0 | 100.0 |
| **36** | Male | 5-6yo | 75.0 | 50.0 | 50.0 |
| **37** | Female | 5-6yo | 100.0 | 75.0 | 100.0 |
| **38** | Male | 5-6yo | 100.0 | 75.0 | 100.0 |
| **39** | Male | 5-6yo | 100.0 | 100.0 | 0.0 |
| **40** | Male | 5-6yo | 100.0 | 100.0 | 0.0 |
| **41** | Female | 5-6yo | 75.0 | 75.0 | 100.0 |
| **42** | Male | 5-6yo | 75.0 | 100.0 | 100.0 |

Agreement (%) with U.S. in Experiment 1b.

| **ID** | **Gender** | **Age group** | **Trustworthiness** | **Dominance** | **Competence** |
| --- | --- | --- | --- | --- | --- |
| **1** | Female | 3-4yo | 71.4 | 100.0 | 60.0 |
| **2** | Female | 3-4yo | 71.4 | 85.7 | 60.0 |
| **3** | Female | 3-4yo | 42.9 | 57.1 | 40.0 |
| **4** | Female | 3-4yo | 28.6 | 100.0 | 60.0 |
| **5** | Male | 3-4yo | 71.4 | 85.7 | 80.0 |
| **6** | Male | 3-4yo | 14.3 | 28.6 | 40.0 |
| **7** | Male | 3-4yo | 100.0 | 14.3 | 60.0 |
| **8** | Female | 3-4yo | 100.0 | 100.0 | 60.0 |
| **9** | Female | 3-4yo | 100.0 | 85.7 | 40.0 |
| **10** | Female | 3-4yo | 100.0 | 100.0 | 60.0 |
| **11** | Female | 3-4yo | 100.0 | 100.0 | 80.0 |
| **12** | Male | 3-4yo | 100.0 | 100.0 | 60.0 |
| **13** | Male | 3-4yo | 71.4 | 100.0 | 40.0 |
| **14** | Female | 3-4yo | 100.0 | 100.0 | 20.0 |
| **15** | Male | 3-4yo | 57.1 | 85.7 | 80.0 |
| **16** | Female | 3-4yo | 100.0 | 85.7 | 60.0 |
| **17** | Male | 3-4yo | 57.1 | 100.0 | 80.0 |
| **18** | Female | 3-4yo | 57.1 | 28.6 | 60.0 |
| **19** | Male | 3-4yo | 100.0 | 0.0 | 20.0 |
| **20** | Female | 3-4yo | 71.4 | 71.4 | 60.0 |
| **21** | Female | 3-4yo | 14.3 | 71.4 | 20.0 |
| **22** | Female | 3-4yo | 100.0 | 100.0 | 80.0 |
| **23** | Male | 5-6yo | 100.0 | 100.0 | 40.0 |
| **24** | Female | 5-6yo | 100.0 | 85.7 | 80.0 |
| **25** | Female | 5-6yo | 100.0 | 100.0 | 60.0 |
| **26** | Male | 5-6yo | 71.4 | 100.0 | 60.0 |
| **27** | Female | 5-6yo | 100.0 | 42.9 | 60.0 |
| **28** | Male | 5-6yo | 100.0 | 100.0 | 60.0 |
| **29** | Female | 5-6yo | 100.0 | 100.0 | 60.0 |
| **30** | Female | 5-6yo | 85.7 | 100.0 | 40.0 |
| **31** | Female | 5-6yo | 57.1 | 100.0 | 80.0 |
| **32** | Female | 5-6yo | 100.0 | 100.0 | 60.0 |
| **33** | Male | 5-6yo | 100.0 | 100.0 | 80.0 |
| **34** | Male | 5-6yo | 100.0 | 100.0 | 40.0 |
| **35** | Male | 5-6yo | 100.0 | 100.0 | 60.0 |
| **36** | Female | 5-6yo | 100.0 | 100.0 | 80.0 |
| **37** | Male | 5-6yo | 57.1 | 100.0 | 100.0 |
| **38** | Female | 5-6yo | 71.4 | 100.0 | 60.0 |
| **39** | Male | 5-6yo | 100.0 | 85.7 | 60.0 |
| **40** | Male | 5-6yo | 100.0 | 85.7 | 60.0 |
| **41** | Male | 5-6yo | 100.0 | 100.0 | 40.0 |
| **42** | Male | 5-6yo | 100.0 | 100.0 | 60.0 |
| **43** | Female | 5-6yo | 100.0 | 100.0 | 80.0 |
| **44** | Female | 5-6yo | 100.0 | 100.0 | 40.0 |
| **45** | Male | 5-6yo | 71.4 | 100.0 | 60.0 |
| **46** | Male | 5-6yo | 100.0 | 85.7 | 60.0 |

Agreement (%) with U.S. in Experiment 2.

| **ID** | **Gender** | **Trustworthiness** | **Dominance** | **Competence** |
| --- | --- | --- | --- | --- |
| **1** | Male | 85.7 | 100.0 | 100.0 |
| **2** | Female | 71.4 | 85.7 | 80.0 |
| **3** | Female | 28.6 | 85.7 | 40.0 |
| **4** | Female | 0.0 | 71.4 | 20.0 |
| **5** | Female | 42.9 | 100.0 | 40.0 |
| **6** | Female | 28.6 | 85.7 | 80.0 |
| **7** | Female | 71.4 | 100.0 | 40.0 |
| **8** | Female | 71.4 | 71.4 | 20.0 |
| **9** | Female | 71.4 | 85.7 | 60.0 |
| **10** | Female | 85.7 | 100.0 | 60.0 |
| **11** | Female | 71.4 | 100.0 | 80.0 |
| **12** | Female | 28.6 | 100.0 | 60.0 |
| **13** | Female | 42.9 | 100.0 | 100.0 |
| **14** | Female | 100.0 | 71.4 | 60.0 |
| **15** | Female | 100.0 | 100.0 | 40.0 |
| **16** | Female | 28.6 | 85.7 | 80.0 |
| **17** | Female | 85.7 | 100.0 | 60.0 |
| **18** | Female | 42.9 | 71.4 | 60.0 |
| **19** | Female | 0.0 | 100.0 | 60.0 |
| **20** | Male | 0.0 | 100.0 | 60.0 |
| **21** | Female | 42.9 | 100.0 | 40.0 |
| **22** | Female | 85.7 | 100.0 | 80.0 |
| **23** | Female | 85.7 | 100.0 | 60.0 |
| **24** | Female | 28.6 | 100.0 | 40.0 |
| **25** | Female | 71.4 | 100.0 | 80.0 |
| **26** | Male | 57.1 | 85.7 | 40.0 |
| **27** | Female | 71.4 | 85.7 | 80.0 |
| **28** | Male | 42.9 | 85.7 | 40.0 |
| **29** | Male | 42.9 | 71.4 | 80.0 |
| **30** | Male | 0.0 | 100.0 | 60.0 |
| **31** | Male | 71.4 | 100.0 | 80.0 |
| **32** | Female | 42.9 | 100.0 | 80.0 |
| **33** | Male | 42.9 | 71.4 | 80.0 |
| **34** | Female | 71.4 | 100.0 | 40.0 |
| **35** | Female | 100.0 | 100.0 | 60.0 |
| **36** | Male | 14.3 | 100.0 | 40.0 |
| **37** | Female | 71.4 | 100.0 | 80.0 |
| **38** | Female | 42.9 | 85.7 | 80.0 |
| **39** | Female | 85.7 | 100.0 | 80.0 |
| **40** | Male | 14.3 | 100.0 | 60.0 |
| **41** | Male | 85.7 | 85.7 | 40.0 |
| **42** | Female | 28.6 | 85.7 | 40.0 |
| **43** | Female | 100.0 | 100.0 | 40.0 |
| **44** | Female | 71.4 | 100.0 | 80.0 |
| **45** | Male | 0.0 | 100.0 | 40.0 |

# Supplementary Tables

**Supplementary Table 1.** Frequencies of the choices in Experiment 1a. “Consistent choice” means the number of the choice consistent with the U.S. Results of the binominal test are indicated as *p* and *g*.

| Trait | Consistent  choice | Inconsistent  choice | *p* | effect size (*g*) |
| --- | --- | --- | --- | --- |
| **Trustworthiness** |  |  |  |  |
| 3-4 years old | 66 | 34 | .00 | .16 |
| 5-6 years old | 56 | 12 | .00 | .32 |
| **Dominance** |  |  |  |  |
| 3-4 years old | 60 | 40 | .06 | .10 |
| 5-6 years old | 52 | 16 | .00 | .26 |
| **Competence** |  |  |  |  |
| 3-4 years old | 28 | 22 | .48 | .06 |
| 5-6 years old | 22 | 12 | .12 | .15 |

**Supplementary Table 2.** Frequencies of the choice in Experiment 1b.

| Trait | Consistent | Inconsistent | *p* | effect size (*g*) |
| --- | --- | --- | --- | --- |
| **Trustworthiness** |  |  |  |  |
| 3-4 years old | 114 | 40 | .00 | .24 |
| 5-6 years old | 155 | 13 | .00 | .42 |
| **Dominance** |  |  |  |  |
| 3-4 years old | 119 | 35 | .00 | .27 |
| 5-6 years old | 160 | 8 | .00 | .45 |
| **Competence** |  |  |  |  |
| 3-4 years old | 61 | 49 | .29 | .05 |
| 5-6 years old | 74 | 46 | .01 | .12 |

**Supplementary Table 3.** Averages of agreement with U.S. [95% CI].

| **Age group** | **Trait** | **Experiment 1a** | | **Experiment 1b** | | **Experiment 2** | |
| --- | --- | --- | --- | --- | --- | --- | --- |
| 3-4 years old | Trustworthiness | .66 | [.55,.77] | .74 | [.64,.84] | .70 | [.62,.78] |
|  | Dominance | .60 | [.49,.71] | .77 | [.67,.87] | .68 | [.62,.75] |
|  | Competence | .56 | [.38,.74] | .55 | [.48,.63] | .56 | [.47,.64] |
| 5-6 years old | Trustworthiness | .82 | [.68,.96] | .92 | [.83,1.02] | .88 | [.80,.97] |
|  | Dominance | .76 | [.63,.90] | .95 | [.86,1.05] | .87 | [.81,.94] |
|  | Competence | .65 | [.43,.87] | .62 | [.54,.69] | .63 | [.54,.72] |
| Adult | Trustworthiness |  |  |  |  | .54 | [.46,.62] |
|  | Dominance |  |  |  |  | .93 | [.86,.99] |
|  | Competence |  |  |  |  | .60 | [.52,.69] |
